# Serum lipid responses to psyllium fiber: differences between pre- and post-menopausal, hypercholesterolemic women

**DOI:** 10.1186/1475-2891-7-22

**Published:** 2008-08-26

**Authors:** Vijay Ganji, Jennifer Kuo

**Affiliations:** 1 Division of Nutrition, School of Health Professions, College of Health and Human Sciences, Georgia State University, Atlanta, GA 30302, USA; 2 Jennifer Kuo, Department of Consumer and Family Studies/Dietetics, San Francisco State University, San Francisco, CA 94132, USA

## Abstract

**Background:**

Cardiovascular disease is the leading cause of death in women and men. Psyllium, a soluble fiber has been known to reduce serum lipids. In this pilot study, we evaluated whether menopausal status would affect the serum lipid responses to psyllium fiber in women.

**Methods:**

Eleven post-menopausal and eight pre-menopausal women with serum total cholesterol >200 mg/dL were included in the study. Subjects consumed their habitual diet and 15 g psyllium/d for 6 weeks. Psyllium was incorporated into cookies. Each cookie contained ≈5 g of psyllium fiber. Subjects ate one cookie in each meal.

**Results:**

With psyllium fiber, total cholesterol concentration was significantly lower (≈5.2%, P < 0.05) in post-menopausal women but not in pre-menopausal women (≈1.3%). Also, there was a significant decrease in HDL-cholesterol in post-menopausal women (≈10.2%, P < 0.05). There were no significant changes observed in concentrations of LDL-cholesterol, triglycerides, apolipoprotein A1, and apolipoprotein B in both pre- and post-menopausal women with psyllium.

**Conclusion:**

In this pilot study, post- and pre-menopausal, hypercholesterolemic women responded differently to psyllium fiber supplementation. Post-menopausal women would benefit from addition of psyllium to their diets in reducing the risk for heart diseases. The results of this study should be used with caution because the study was based on a small sample size.

## Introduction

Cardiovascular disease (CVD) is the leading cause of death among women and men. About 13 million Americans have CVD and approximately 450,000 people die each year in the US from CVD [1]. Risk factors for CVD in women include post-menopausal status, age, hypertension, smoking, and diabetes [2]. The onset of menopause coincides with elevated serum lipids such as total cholesterol, LDL-cholesterol, and triglycerides and decreased HDL-cholesterol [3]. Elevated serum lipids in post-menopausal women are partly due to the loss of estrogen [4]. Hormone therapy showed a reduction in LDL-cholesterol and an increase in HDL-cholesterol [5]. It also frequently increased triglycerides by increasing the production of triglyceride-rich VLDLs [6]. Additionally, hormone therapy increased the risk for stroke, breast cancer, and cholecystitis [7]. Hormone replacement therapy is not for all post-menopausal women [8].

Generally, relative to drug therapy, dietary intervention is less expensive and cost effective for primary intervention of heart diseases [9]. Consumption of foods containing dietary fiber, may improve the long-term maintenance of low atherogenic LDL-cholesterol [10]. Psyllium (*Plantago ovata*) seed husk fiber, a widely used soluble fiber, has been known to reduce serum total cholesterol and LDL-cholesterol [11-13]. Additionally, psyllium fiber supplementation with 10 mg of simvastatin (hypocholesterolemic drug) was as effective as 20 mg of simvastatin alone [14]. This suggests that psyllium fiber is an effective adjuvant hypocholesterolemic agent.

There is a paucity of data on how fiber therapy modulates the risk for CVD in women. Previous studies conducted in women indicated that dietary therapy for lowering serum lipids differs according to menopausal status [15]. In men compared to post-menopausal women, a greater decrease was observed in total cholesterol after 4 months of high fiber diet [16] suggesting that gender differences exist in hypocholesterolemic effect of dietary fiber. Limited studies were conducted comparing lipid responses to psyllium fiber intervention in pre- and post-menopausal women. Therefore, we tested the hypothesis of whether serum lipid responses to psyllium fiber intake in post-menopausal, hypercholesterolemic women differ from pre-menopausal, hypercholesterolemic women.

## Methods

### Subjects

A total of 25 hypercholesterolemic women (13 pre-menopausal and 12 post-menopausal) were recruited for the study. Hypercholesterolemia was defined as having serum cholesterol >200 mg/dL. Subjects were recruited from San Francisco and surrounding communities through posting of flyers. Participants lived in their homes during the study. The exclusion criteria were smoking, diabetes, pregnancy, breast feeding, allergic to psyllium fiber, and blood cholesterol ≤200 mg/dL. Individuals with thyroid and gastrointestinal diseases, CVD, chronic alcohol and drug abuse, individuals who were taking lipid-lowering and/or anti-hypertensive medications, and individuals who were on estrogen replacement therapy were excluded from the study. Also, strict vegetarians (vegans) were excluded from the study because their habitual fiber intake is generally higher than non-vegetarians. Post-menopausal status was defined as women who were into menopause at least one year from their last menstruation at the beginning of the study. Subjects were asked to continue their habitual diet and to maintain their pre-study physical activities and body weights. Study protocol and informed consent were approved by the Human Subjects Protection Committee at San Francisco State University.

### Administration of psyllium fiber

Subjects were asked to consume psyllium fiber enriched cookies for 6 weeks. We incorporated 15 g of psyllium fiber into cookies (≈5 g/cookie). Subjects consumed 3 cookies/d (one in each meal). Psyllium fiber enriched cookies were made in the departmental food laboratory, according to the formulation given in Table 1. Previous studies documented that 4–6 week feeding period was adequate to observe changes in serum lipids with psyllium fiber intake [17,18]. The reason that psyllium fiber was incorporated into cookies rather than administering as supplements because fiber has shown to produce maximum cholesterol-lowering effect when it is taken mixed with foods [19]. Numerous products (muffins and powdered drink mixes) incorporating psyllium were tested for palatability and cookies yielded the most appetizing product. Cookies were individually supplied to subject's home in a fresh or frozen form on a weekly basis. The rationale for the amount of fiber used (15 g/d) was based on the recommendation of 25–35 g/d of fiber intake for adults [20]. Since Americans consume about 15 g/d of fiber [11], by adding 15 g/d subjects would meet the recommendations for fiber. During the study, subjects were asked to refrain from consuming fiber supplements. If a subject was a social drinker that subject was asked to maintain her usual alcohol intake level.

**Table 1:** Composition of psyllium fiber-enriched cookies*

Ingredients	Amount^#^
Psyllium fiber, g	250
All purpose flour, g	252
Sugar, g	294
Brown sugar, g	316.5
Shortening, g	484
Eggs, g	200
Baking soda, g	4.68

Vanilla extract, mg	3.5

* = Procedures: Mixed dry and wet ingredients well. Weighed the dough and divided by 50. Baked at 330°F for 11–14 minutes until cookies became light golden brown. # = Made 50 cookies. Each cookie contained approximately 5 g of psyllium fiber.

### Serum lipid analysis

Twelve-hour overnight fasting blood samples were obtained from venipuncture in the arm at the beginning and at the end of the 6-week period by a certified phlebotomist. Blood samples were centrifuged and serum was separated before lipid analysis. Serum samples were analyzed for total cholesterol, LDL-cholesterol, HDL-cholesterol, triglycerides, apolipoprotein A-1, and apolipoprotein B using an automatic analyzer (Quest Diagnostic Laboratories, Dublin, CA).

### Data analysis

Data were presented as mean ± standard deviation. Paired t-test was used to determine the significant difference between the baseline and the post-fiber treatment values in pre- and post-menopausal women (Microsoft Excel for Windows, 1999). Statistical significance was set at P < 0.05.

## Results

Of 25 subjects, 19 (8 pre- and 11 post-menopausal) women completed the study. Reasons for discontinuation of participation included gastrointestinal discomfort, increased frequency of bowel movements, softer stools, and personal travel and time conflicts. Subjects' characteristics were summarized in Table 2. As expected, the post-menopausal women were significantly older compared to pre-menopausal women (p < 0.05). Body mass index (BMI) at baseline between pre- and post-menopausal women was not significantly different. BMI for all subjects were within the desirable low health risk range. There were no significant differences in body weight between baseline to post-study measurements in both pre- and post-menopausal women (data are not shown).

**Table 2:** Characteristics of subjects*

Variable	Pre-menopausal women	Post-menopausal women
Race-ethnicity		
White/Hispanic/Asian, n	1/1/6	0/0/11
Age, y	34.6 ± 11.5	52.9 ± 2.8^#^
Weight, kg	63.2 ± 9.3	57.6 ± 5.6^@^
Height, m	1.6 ± 0.1	1.6 ± 0.04^@^

BMI, kg/m^2^	24.2 ± 4.2	22.6 ± 2.2^@^

* = Mean ± standard deviation. ^# ^= Significant difference from pre-menopausal women (p < 0.05).

^@ ^= No significant difference from pre-menopausal women.

The baseline and post-study serum lipid concentrations for pre- and post-menopausal women are presented in Table 3. Mean serum total cholesterol concentration with psyllium fiber intake was significantly lower compared to baseline values in post-menopausal women (218.7 mg/dL vs. 230.8 mg/dL; p < 0.05) but not in pre-menopausal women (240.5 mg/dL vs. 243.6 mg/dL). The decrease in post-menopausal women with respect to total serum cholesterol was ≈5.2%.

**Table 3:** Serum lipid responses to psyllium fiber intake in pre- and post-menopausal women*

Serum lipid	Pre-menopausal women (n = 8)	Post-menopausal women (n = 11)
Total cholesterol		
Baseline, mg/dL	243.6 ± 40.3	230.8 ± 24.4
Post-fiber, mg/dL	240.5 ± 41.2	218.7 ± 17.0^#^
LDL-cholesterol		
Baseline, mg/dL	156.0 ± 46.0	133.4 ± 32.3
Post-fiber, mg/dL	151.0 ± 42.0	127.3 ± 14.3
HDL-cholesterol		
Baseline, mg/dL	61.8 ± 12.2	65.0 ± 17.0
Post-fiber, mg/dL	61.4 ± 24.1	58.4 ± 14.8^#^
Triglycerides		
Baseline, mg/dL	129.5 ± 52.1	139.5 ± 72.1
Post-fiber, mg/dL	139.4 ± 77.4	165.4 ± 85.2
Apolipoprotein A-1		
Baseline, mg/dL	152.5 ± 30.3	150.6 ± 21.8
Post-fiber, mg/dL	154.4 ± 39.2	152.3 ± 23.8

Apolipoprotein B		
Baseline, mg/dL	122.3 ± 34.7	110.6 ± 19.7
Post-fiber, mg/dL	117.1 ± 31.5	106.3 ± 16.5

* = Mean ± standard deviation. # = Significantly different from pre-menopausal women, paired t-test (p < 0.05).

Mean HDL cholesterol was significantly lower in post-menopausal women with fiber intake (65 mg/dL to 58.4 mg/dL) (p < 0.05). In contrast, no change was observed in HDL-cholesterol concentrations in pre-menopausal women with psyllium. Changes in LDL-cholesterol in pre- and post-menopausal women from baseline to post-study were not significant although there was a slight decline in values with psyllium fiber intake. The decline was ≈4.6% in post-menopausal women and was ≈3.2% in pre-menopausal women.

There was a ≈7.6% and ≈18.6% increase in serum triglycerides in pre- and post-menopausal women, respectively, with psyllium fiber supplementation. However, these changes were not statistically significant. Similarly, there were no significant changes in response to psyllium fiber intake in serum apolipoprotein A-1 and apolipoprotein B concentrations in both pre- and post-menopausal women.

## Discussion

We investigated the effect of psyllium fiber intake at 15 g/d dosage level for 6 weeks on serum lipids in free-living, pre- and post-menopausal, hypercholesterolemic women. Psyllium fiber significantly lowered serum total cholesterol in post-menopausal women. Decreased total serum cholesterol was primarily due to changes in the HDL-cholesterol. Although LDL-cholesterol was lower (≈4.6%) with fiber intake, the decrease was not statistically significant when compared to baseline values in post-menopausal women. Concentrations of serum triglycerides, apolipoprotein A-1, and apolipoprotein B were not affected by psyllium fiber intake in post-menopausal women. Also, psyllium fiber intake had no effect on serum total cholesterol, LDL-cholesterol, HDL-cholesterol, triglycerides, apolipoprotein A-1, and apolipoprotein B in pre-menopausal, hypercholesterolemic women.

Previously, we documented cholesterol-lowering effect of psyllium in normocholesterolemic humans [18]. Other studies have shown that consumption of psyllium fiber reduced serum total cholesterol and LDL-cholesterol in hypercholesterolemic subjects [12,13]. Several other investigators reported a 3–17% reduction of serum total cholesterol and a 4–20% reduction of LDL-cholesterol with psyllium fiber [12,19,21,22]. In a meta-analysis, Anderson et al. (2000b) reported that consumption of 10.2 g psyllium/d lowered total cholesterol by 4%. Whereas, in our study, we achieved a ≈5.2% decrease in total serum cholesterol concentrations at a dosage level of 15 g psyllium/d. Most of the psyllium fiber used in previous studies was either in the form of ready-to-eat cereal or as fiber supplement. In our study, we administered psyllium fiber in cookies. Thus, the method of administration of psyllium might account for some differences in cholesterol-lowering property of psyllium fiber in various studies.

Davidson et al. [10] reported that consumption of psyllium fiber lowered total cholesterol without modifying HDL-cholesterol concentrations. In contrast, our study did not reveal that psyllium fiber reduced serum total cholesterol without lowering HDL-cholesterol. The discrepancy in results between this study and other studies may likely be due to differences in characteristics of subjects. Also, our subjects consumed self-selected diets during the study. Thus, the diet intake might have some influence on the study outcomes.

In this study, we found the hypocholesterolemic effect of psyllium fiber in post-menopausal women but not in pre-menopausal women indicating that pre- and post-menopausal women responded differently to psyllium fiber. Lack of significant hypocholesterolemic effect of psyllium fiber in pre-menopausal women suggests that the elevated cholesterol concentrations in those subjects might be due to genetic causes and thus are unresponsive to dietary modification. On the other hand, elevated cholesterol in post-menopausal women is often due to deranged lipid metabolism (non-genetic causes) arising from loss of estrogen function and thus is responsive to psyllium fiber intervention. In post-menopausal women with established CVD, higher intake of fiber has been associated with less progression of coronary atherosclerosis [23]. Also, psyllium fiber significantly reduced post-prandial concentrations of serum triglycerides, glucose, and insulin in persons with diabetes [24] suggesting a role for psyllium fiber in the management of diabetes.

Previous studies on the effect of psyllium fiber on triglycerides yielded equivocal results [22,25,26]. Psyllium fiber intake significantly reduced plasma triglycerides in men, but in post-menopausal women, it resulted in a significant increase with no change in pre-menopausal women [26]. In our study, we found no significant effect of psyllium fiber intake on serum triglycerides in both pre- and post-menopausal women, although there was a trend towards increased serum triglycerides.

The lack of effect of psyllium fiber intake on serum lipids in pre-menopausal women might be due insufficient number of subjects recruited to observe statistically significant differences. Large sample size might reveal significant total cholesterol and/or LDL-cholesterol reduction. Therefore, the results of this study must be used with caution. Although, we have instructed the subjects not to alter their dietary habits during the study, it is possible that our results may have confounded by the fiber from unintended food sources. Because psyllium fiber was incorporated into cookies, consumption of psyllium enriched cookies lead to an additional intake of ≈30 g/d fat. This may have blunted the hypocholesterolemic effect of psyllium in both pre- and post-menopausal women. Additionally, all subjects in the post-menopausal group were Asians. It is not known how this has affected the cholesterol-lowering effect of psyllium.

In conclusion, post- and pre-menopausal women differed with regard to lipid responses to psyllium fiber. Serum total cholesterol was responsive to psyllium fiber in post-menopausal women. Post-menopausal women would likely benefit from addition of psyllium fiber to their diets in reducing the risk of heart diseases because every 1% reduction in serum total cholesterol concentration results in 2% reduction in risk for heart diseases [27]. Differences in cholesterol-lowering response to psyllium intake may be related to the differences in hormonal status between pre- and post-menopausal women [15,26]. Additionally, consumption of psyllium fiber provides a low-cost adjunct to the National Cholesterol Education Program diets for hypercholesterolemia.

## Competing interests

The authors declare that they have no competing interests.

## Authors' contributions

VG and JK directed the research. VG was responsible for getting the support for the project. Both authors are responsible for collecting the data, analysis of data, and drafting the manuscript. All authors read and approved the manuscript before submission.
